# Hypnosis in psychotherapy, psychosomatics and medicine. A brief overview

**DOI:** 10.3389/fpsyg.2024.1377900

**Published:** 2024-03-22

**Authors:** Burkhard Peter

**Affiliations:** ^1^MEG-Foundation, Wilhelmsthal-Hesselbach, Germany; ^2^School of Dental Medicine, University of Bern, Bern, Switzerland

**Keywords:** clinical hypnosis, psychotherapy, psychosomatics, medicine, imagination, relaxation, hypnotizability, effectivity

## Abstract

Aspects of hypnosis and its application in psychotherapy, psychosomatics and medicine are examined and contextualized in the 250-year history of hypnosis. Imagination as an essential element of hypnotic treatments appeared as early as 1784 as an argument rejecting the theory of animal magnetism of Franz Anton Mesmer. In somnambulism of German romanticism, another proto-form of hypnosis after 1800, concepts of the mind–body problem were dealt with, which still characterize the understanding of unconscious mental processes today. Hypnosis was at the beginning of psychoanalysis, but was not pursued further by Sigmund Freud from 1900 onwards. Nevertheless, there were some hypnoanalytical approaches in the 20th century, as well as attempts to integrate hypnosis into behavior therapy. Techniques of imagination and relaxation combine both; in particular findings from cognitive psychology explain processes of both hypnosis and cognitive behavioral therapy. The influence of social psychology brought a new perspective to the debate about the nature of hypnosis, which continues to this day: is hypnosis to be understood as a special state of consciousness or is it a completely normal, mundane interaction? The experiments that were carried out to support one side or the other were also dependent on the hypnotizability of the subjects involved, as the more difficult hypnotic phenomena such as paralysis, hallucinations or identity delusions can only be demonstrated by highly hypnotizable subjects. The fact that these are not mere compliance reactions has now been proven by many studies using imaging techniques. But even those who are moderately hypnotizable benefit from hypnosis rituals. Variables postulated by socio-cognitive hypnosis researchers, such as motivation and expectation, are relevant, as is a good “hypnotic rapport.” Practical application of hypnotherapy today is characterized by the innovative techniques and strategies developed by Milton H. Erickson. Research into the effectiveness of hypnosis in the field of psychotherapy and psychosomatics still leaves much to be done. The situation is different in the field of medical hypnosis, where there are considerably more studies with a satisfactory design and verifiable effects. However, the impact in practical application in everyday medical practice is still low. Newer developments such as virtual reality and artificial intelligence are being looked at with critical interest.

## 1 Introduction

The history of hypnosis in modern era goes back almost 250 years. Based on this historical development, some key topics of clinical hypnosis and hypnotherapy are discussed. Due to the author’s profession, the view on the subject is primarily that of a hypnotherapist and trainer in clinical hypnosis.

The “domain of hypnosis” (Hilgard, 1973) is usually divided into experimental and clinical hypnosis. Experimental hypnosis is basic research into the nature of hypnosis and its phenomena. As this is not the focus of this article, only some of the results will be mentioned. Clinical hypnosis refers to the use of hypnotic trance and hypnotic phenomena in the fields of psychotherapy, psychosomatics and medicine (including dentistry). These are the main topics of this article. The use of hypnosis in forensic settings hardly plays a role any more (Reiser, 1980; Beetz and von Delhaes, 2023). The topics of stage hypnosis (e.g., Kleinhauz et al., 1984) and contraindications (Revenstorf and Peter, 2023) are also touched on in passing.

Hypnosis cannot be seen independently of suggestion. Hypnosis is defined here as an intra-personal state of consciousness, suggestion as an act of inter-personal communication (Peter, 2024). The “correct” understanding of these two terms and their relationship to each other has been the subject of an ongoing debate for almost 250 years, which has not facilitated the acceptance of clinical hypnosis, especially among science-oriented researchers and clinicians who dominate today’s human sciences of medicine and psychology.

## 2 Hypnosis in psychotherapy and psychosomatics

### 2.1 Hypnosis and the unconscious

The German-American hypnoanalyst Erika Fromm described hypnosis as the “royal road to the unconscious” (Fromm, 1992): hypnosis is suitable for uncovering “unconscious” information from a patient’s life history, i.e., information that is not accessible to the conscious mind, in order to gain relevant insights into the etiology of a disorder and thus resolve unconscious psychodynamic conflicts. Wolberg (1945) had already extended this purely explorative hypnoanalytic procedure by adding corrective new experiences to the patient’s experiential reality, so that symptoms could disappear because their function had become obsolete or because the patient’s self-efficacy had changed significantly. However, this therapeutic strategy of “reconstructing the past” had already been practiced by Janet (1889), but was hardly recognized in the “golden age of hypnosis” at the end of the 19th century, especially not by his contemporary Sigmund Freud who is often referred to as the discoverer of the unconscious. In fact, the recognition of the role of the unconscious precedes Sigmund Freud: the adjective “unconscious” can already be found at the end of the 18th century, among others in Schiller and Goethe (Goldmann, 2005). The term “the unconscious” gained particular significance—especially in connection with the proto-hypnosis, the animal magnetism of Mesmer (1779)—in the work of the German philosopher Schelling (1800). Schelling’s philosophy of nature helped orthodox magnetism, which had almost been forgotten by 1800, to assume importance in the shape of romantic somnambulism. For Romantics, the latter confirmed their view of the world as animated by a world spirit (Weltgeist) or world soul (Weltseele). This offered explanations for the fantastic phenomena and abilities that were exhibited by some patients in a state of magnetic somnambulism and fascinated many doctors, but also artists and educated people during the period of German Romanticism between 1800 and 1848 (Gauld, 1992; Peter, 2009, 2023c). It is noteworthy that in this period of Romantic medicine, the symptoms of somnambulism were regarded as special talents; they could occur spontaneously or be artificially induced by the technique of “mesmerisation” and gave those affected a special aura and occasionally national fame, such as the “seeress of Prevorst” (Kerner, 1829). However, some of the many reports from that time can be read today as detailed descriptions of severe psychopathologies such as dissociative personality disorders (Peter, 2011).

Almost at the same time as Schelling’s philosophical ideas, the physiological prerequisites for the unconscious were also discovered by the Berlin medical professor Johann Christian Reil. Reil’s neurophysiological system of a polar arrangement of “cerebral and ganglionic systems”—analogous to our modern “central and autonomic nervous system”—was a precursor model for the later ideas of a psychodynamic mind–body connection as early as 1807: “Consciousness” and the “thinking soul” (Reil, 1807) are localized in the “cerebral system” based in the brain. The “ganglionic system” is the seat of the vegetative, the passions, the sentient soul and the “unconscious ideas” (p. 212). Both systems are connected by an “apparatus of semiconduction,” which isolates them from each other in the normal waking state, but creates a good connection in states of somnambulism (p. 192). Reil’s physiological model resonated with the natural philosophical followers of magnetic somnambulism, who began to formulate the history of the unconscious and of psychosomatic connections.

However, the understanding of unconscious mental processes and their influence through the proto-hypnosis techniques of “magnetising” or “mesmerising” differed fundamentally from the original theory of Franz Anton Mesmer (1812) in the period of romantic somnambulism at the beginning of the 19th century, who saw animal magnetism as a physical rather than a psychic force. To regard Mesmerism as the forerunner of what we understand today as hypnosis and hypnotherapy, as is traditional in the Anglo-American hypnosis literature following Ellenberger (1970), can therefore certainly be questioned (Peter, 2005).

Sigmund Freud—like so many of his medical contemporaries at the end of the 19th century—had become acquainted with hypnosis in France, with Charcot in Paris and with Bernheim in Nancy, from which he received decisive impulses for his insights into the unconscious nature of human beings (Chertok, 1968a). However, through the influence of Schopenhauer and Nietzsche, his view of the unconscious had changed decisively from the exclusively positive connotation of romantic somnambulism to a partly dystopian entity, a place of repressed problematic affects or even destructive drives such as Thanatos. Hypnosis no longer played a role for Freud in the development of his psychoanalysis from 1900 onwards, apart from the necessities towards the end of the First World War and his many war neurotics:

“It is very probable, too, that the application of our therapy to numbers will compel us to blend the pure gold of analysis plentifully with the copper of direct suggestion and hypnotic influence could also find a place there again, as in the treatment of war neurotics […].” (Freud, 1919, p. 402)

There were already some hypnoanalytic approaches at that time (Schilder and Kauders, 1926; Lifschitz, 1930). Essentially, however, hypnosis developed in the first half of the 20th century independently of or parallel to psychoanalysis (Peter and Lenhard, 2016) as hypnotic suggestive therapy according to the guidelines of the French school of Bernheim (1886), in which “the unconscious” had lost its central meaning and had been replaced by the term “subconscious,” understood as a semantic store of problematic beliefs and convictions that had to be corrected by persuasive suggestions given after a hypnosis induction.

It was the American psychiatrist and psychotherapist Milton H. Erickson who reintroduced the word “unconscious” into hypnosis, in its original Romantic meaning as a metaphor for a patient’s positive resources that can be used to overcome problems and strengthen self-efficacy. Thus “the unconscious” became a metaphorical figure for a moderating “therapeutic tertium” (Peter, 2002) in the interaction between therapist and patient. In Ericksonian hypnosis and psychotherapy, the special or non-ordinary state of consciousness of the hypnotic trance was no longer understood merely as a “sedative for the conscious mind” (Peter, 2009) in order to allow the therapeutic suggestions to have an unhindered influence on the patient’s “subconscious”—i.e. “to slide the suggestion underneath the patient” (literal translation of the Latin verb “sub-gerere”), so to speak—as still assumed in classical suggestive hypnosis à la Bernheim, but as a possibility of direct communication with the “unconscious,” e.g., via ideomotor signaling (Cheek, 1962b; Peter, 2023e). Arm levitation (Peter et al., 2012) or finger signaling, for example, make it possible to initially make contact with the “unconscious” non-verbally in order to activate episodic content from the patient’s past experience or reactions stored in the patient’s body memory, which can then be described verbally and made accessible for cognitive processing. “Uncovering” unconscious conflicts without hypnosis was and is also the goal of psychodynamic psychotherapy, but it takes a long time. Hypnosis facilitates and accelerates this process—according to the arguments of hypnoanalysts following Erika Fromm (1965). However, Erickson’s new idea—although old in relation to romantic somnambulism—was to attribute positive characteristics and abilities (positive resources) to the unconscious, which are crucial for therapeutic progress. The classical idea, already contained in suggestive hypnosis, that hetero-hypnotic suggestions, i.e., suggestions presented by the therapist, can only be effective if they are accepted and implemented by the patient auto-hypnotically, was elaborated in a much more differentiated way by Milton H. Erickson. In addition to the many other innovative ideas that Erickson introduced into psychotherapy and hypnotherapy, his emphasis on patient-centeredness and resource orientation from the 1970s onwards brought a remarkable innovation for hypnosis which was now clearly different from the “old” authoritative suggestive hypnosis.

Sigmund Freud had learnt about this “old school” hypnosis from Bernheim in 1889 and later understandably abandoned it in the course of developing his psychoanalysis from 1900 onwards. However, he had previously used it and later referred to it favourably from time to time, e.g.:

“We must still be grateful to the old hypnotic technique for having brought before us single psychical processes of analysis in an isolated or schematic form. Only this could have given us the courage ourselves to create more complicated situations in the analytic treatment and to keep them clear before us.” (Freud, 1914, p. 148)

The classic example of the special possibilities of hypnosis to intervene in unconscious psychodynamic processes is the “cathartic” therapy of Berta Pappenheim (Anna O.) from the “Studies on Hysteria” by Breuer and Freud (1895): Only under hypnosis did the patient remember stressful traumatic situations and was able to report them in detail in the “talking cure” as well as reassociate the split-off affects. Chertok (1961) has explained why this therapy of Anna O. was not successful, but led Freud to the development of one of the most important concepts of psychoanalysis, namely that of transference (Chertok, 1968b). Similar “hypnoanalytic” procedures that were successful can later be found in many reports from the 20th century (Wolberg, 1948; Watkins, 1992).

### 2.2 Hypnosis and cognitive psychology

Without reference to the historical sources mentioned above, Alldredge and Elkins (2023) have recently presented a version of Epstein’s (2014) cognitive-experiential self-theory adapted to hypnosis, but which can also be found in a similar form in other contemporary concepts: Evans (2011) dual-process theory distinguishes between type 1 mental processes, which are intuitive, fast and largely automatic, and the slower, reflexive, analytical and cognitive processes of type 2, which also utilize working memory, as well as Kahnemann’s (2011) description of the two modes of “thinking, fast and slow.” It has also been pointed out in classical cognitive psychology (Paivio, 1971; Lang, 1979; Tulving, 1985), with reference to hypnosis also by Kihlstrom (1987) or more recently by Landry et al. (2014) that there are different forms of encoding information. Put simply, verbally encoded information corresponds to narrative memory, i.e., the content of declarative memory or explicit knowledge (“factual knowledge”). This initially appears to be one domain of cognitive behavior therapy, not so much that of hypnotherapy. But verbally encoded information can also be non-conscious, can be subject to all forms of cognitive distortion or can be state- and context-dependent; in this case, re-experiencing the corresponding original psychophysiological state in hypnosis is a way of uncovering deeply rooted beliefs and changing them with the help of associative-divergent thinking. Many personal and especially problematic or traumatic experiences are not verbally coded, but are stored directly in episodic and/or procedural memory stores (“experiential knowledge” and “body memory” or “embodied”) and therefore have an influence on a patient’s symptoms. Although the aim of other methods such as psychoanalysis (e.g., by free association) or cognitive behavioral therapy (e.g., by Socratic dialogue) is to make the patient understand such connections and thereby resolve symptoms, hypnosis makes it easier to access these memories and accelerates this process. One possible explanation for this is that the induction of a hypnotic trance favors a state of sensory deprivation and motor restriction (Peter, 1994, 2023e),^1^ which constricts general attention and peripheral awareness and focuses on the essential content of the suggestions. This makes it easier to address these other functional units of perception and consciousness, which are only active in the background or subliminal in the usual patterns of everyday consciousness, i.e., unnoticed or “unconscious.”

Another classic example, which differs significantly from the above-mentioned “Anna O.,” was described by Janet (1889) with his patient Marie. In a hypnotic trance, he initially led her back to the experience of symptom genesis. However, instead of merely reporting the traumatic situation and allowing the affect to be reacted to, as with Breuer and Freud (1895), he enabled the patient to experience new representations of parts of her past quite vividly and evidently in hypnotic age regression:

“At the age of 13 Marie had had her first menstruation, but because of some childish notion or something she had heard and misunderstood, she thought it was something shameful, and she devised a means of stopping the bleeding as quickly as possible. About 20 hours after the bleeding started, she secretly went out and sat in a large bucket of cold water. The success was complete; the menstruation suddenly stopped and although she got severe chills, she was just able to manage the journey home. She was ill for quite a long time and was delirious for several days. But everything got back on track and menstruation did not return until five years later. When it came again, it brought the disorders with it [namely pain, nervous cramps, trembling all over the body and then long and severe delirium]. […] But as I now had more time at my disposal, I tried again; I only succeeded by an unusual means. It was necessary to restore Marie by suggestion to the age of 13, to bring her back to the initial circumstances of the delirium, to convince her that the menstruation had already lasted three days and had not been interrupted by any unfortunate event. As soon as this was done, the following menstruation occurred at the proper time and lasted for three days without any pain, cramps or delirium.” (Janet, 1889, p. 435)

Similar cases of such “reconstructions of the past” carried out under hypnosis can be found in Wolberg (1948), Erickson and Rossi (1989) or Peter (2023b). In contrast to a naïve interpretation of the supposedly omnipotent helpful possibilities of the unconscious, as can be found in hypnotic lay healers, these hypnotherapists used their therapeutic expertise acquired through study and training to convey new information to their patients and actively help them to have new experiences. In such and similar cases, the hypnotic trance has the function of giving these new experiences the character of reality, i.e., making them evident in the form of hallucinations or illusions (Peter, 2015a,b). With reference to Janet (1894), but without inducing a hypnotic trance, i.e., as a purely imaginative procedure, cognitive behavior therapy has adopted this technique as “imagery rescripting” (Arntz, 2011).^2^

The realization that the experience of reality is heightened in hypnosis and that it is therefore not possible to distinguish within the hypnotic context whether the experience of past events is actually “recovered” memories (bottom-up) or only suggested “false” memories (top-down) would have helped in the 1990s not only to avoid the nonsensical dispute that had been carried out mainly between many trauma therapists and researchers (Yapko, 1994a) and had lasted for a long time (Patihis et al., 2014), but also the suffering brought to many families by allegations of sexual abuse supposedly „uncovered” in trauma therapies (Yapko, 1994b; Brown et al., 1998). The possibility of paramnestic phenomena such as suggested pseudo-memories is precisely the prerequisite for new constructions (of parts) of the past in hypnosis. However, this can lead to false accusations if real third parties are involved and it actually led to the „war of rememberance” (Fried, 1994). This topic has been intensively researched in those years (e.g., Loftus, 1997).

### 2.3 Hypnosis and imagination

Mesmer’s attempt to have his discoveries scientifically evaluated in Paris in 1784 ended with the expert opinions of two scientific commissions stating that the phenomena exhibited by his patients were due to imagination, not to the workings of the animal magnetism he postulated: “The violent symptoms observed in the public exhibition are to be ascribed to […] the imagination called into action” (Franklin et al., 1784, p. 126). So, if magnetism was not needed at all back then, but imagination was sufficient to show the magnetic phenomena, is hypnosis as a “special state of consciousness” necessary today to show hypnotic phenomena, as the group of consciousness researchers around Hilgard (1977) have tried to prove, or is imagination really enough on its own? This was obviously the opinion of the American hypnosis researcher Theodore X. Barber who only wrote the word “hypnosis” in quotation marks from the 1960s onwards. With his book “Hypnosis: A scientific approach” (Barber, 1969), he laid the foundation for an entire generation of researchers who were no longer concerned with the intrapsychic variable of a state of consciousness altered by hypnosis—and certainly not with “the unconscious”—but with complex socio-psychological and socio-cognitive variables such as social interaction, role enactment, attitude, motivation or expectation (e.g., Barber and Calverley, 1962; Coe, 1966; Kirsch, 1985; Spanos, 1991). Consequently, he left it open whether at the beginning of his “Creative Imagination Scale” (CIS) (Wilson and Barber, 1978), with which he tested the suggestibility of his subjects, a classic hypnosis induction was presented as in the traditional Stanford or Harvard scales of hypnotic susceptibility (SSHS; HGSHS) (Weitzenhoffer and Hilgard, 1959; Shor and Orne, 1962) or only a short text such as the following: “These are all tests of imagination. The better you can imagine and the harder you try, the more you’ll respond. Try as hard as you can to concentrate, and to imagine the things I tell you” (Barber and Glass, 1962). The subsequent test items refer to the same hypnotic phenomena as in the Stanford and Harvard scales (Peter, 2024). Barber and his successors thus demonstrated that a “hypnotic” state is not required to show “hypnotic” phenomena. But why do we still need hypnosis if “guided imagining” (Barber and Wilson, 1979) is sufficient?

It is obvious that imagination plays a major role in hypnosis (Wilson and Barber, 1982; Kunzendorf et al., 1996), but the relationship is complex (Sheehan, 1979; Sheehan, 1995): There are for example highly hypnotizable people who cannot imagine at all, and there are people with strong imaginative abilities who have little or no hypnotizability. For example, there is a group of highly hypnotizables who are not characterized by rich imaginative activity in a hypnotic trance, but rather by a great tendency to dissociate (Barrett, 1996; Terhune et al., 2010; Peter et al., 2014). The latter tend to be regarded as difficult patients in therapy—they are often the more vulnerable or even traumatized. The highly imaginative, on the other hand, are usually perceived as easily hypnotizable, cognitively flexible and creative. In addition, there is now also neurophysiological evidence (Oakley and Halligan, 2013) “that mental representations that are produced by voluntary acts of imagination are different from those resulting from hypnotic suggestion [… i.e. …] responses to hypnotic suggestions among highly suggestible individuals are independent of imagery and imagination” (Terhune and Oakley, 2020, p. 722). Moreover, McConkey et al. (1979) and Laidlaw and Large (1997) found that the CIS correlates well with the HGSHS, but that the two tests are independent of each other in their underlying dimensions.

Nevertheless, if the “induction of a hypnotic state” is not considered necessary, but the “instruction to imagine” should be sufficient, then in the sense of T.X. Barber, “hypnotic” imagination is a suitable instrument for behavior therapy, especially after its “cognitive turn.” It is therefore not surprising that many works on this topic were published between the 1970s and 1990s, e.g., by Clarke and Jackson (1983), Dengrove (1976) or Peter et al. (1991). Before that, however, Cautela (1966a,b) had pointed out that systematic desensitization had nothing to do with hypnosis, and another “father of behavior therapy,” Joseph Wolpe (1996), candidly described his development away from hypnosis towards behavior therapy. Weitzenhoffer (1972) compared behavioral and hypnotherapeutic techniques, Lazarus (1973) considered hypnosis as a facilitator in behavior therapy, Spanos (1976) described the “common denominators” of the two methods, Ascher (1977) “the role of hypnosis in behavior therapy,” Kraiker (1985) “cognitive models of hypnotic phenomena” and “The birth of behavioral therapy from the spirit of hypnosis” (Kraiker, 1987), Peter (1992) the many, purely behavioral exposure therapies of Erickson, and Spinhoven (1987) and Humphreys (1986) carried out extensive reviews—to name just a few of the numerous works that linked hypnosis with behavior therapy. Kirsch et al. (1995) conducted the first large meta-analysis for this period (1971–1993) and found that cognitive-behavior therapy treatments in which hypnosis was used additionally showed an effect size almost twice as high as cognitive-behavior therapy treatments without hypnosis. Ramondo et al. (2021) carried out an update after 25 years and were able to replicate the results: Hypnosis increases and prolongs treatment outcomes of cognitive-behavior therapy.

Cognitive behavior therapy has evolved and now makes extensive use of imaginative techniques, without referring to them as “hypnotic,” either with or without inverted commas. Imagination is one of the two techniques with which Wolpe (1961) introduced systematic desensitization at the beginning of behavior therapy. The other technique is progressive muscle relaxation (Jacobson, 1929), which he found more suitable than hypnosis (Wolpe, 1996). The advantage of both techniques, imagination and muscle relaxation, is obvious: they can be carried out arbitrarily and can therefore be taught by instruction. They do not aim to induce a “different,” i.e., hypnotic, state of consciousness in order to suggest involuntary behavior or even the illusion or hallucination of an “alternative reality” (Peter, 2015b); they can therefore neglect the patient variable of hypnotizability (see below) and can therefore be used with significantly more patients than the original suggestive-hypnotic techniques. The adjective “hypnotic” can also be avoided, which is an advantage because of the negative connotation it still has for some—but regrettable for other patients because the expectation effect it creates cannot be utilized (Kirsch, 1985). After all, one of the starting points for the above-mentioned meta-analysis by Kirsch was the following consideration:

“Typical hypnotic inductions closely resemble conventional relaxation training. In fact, all that is needed to convert relaxation training into a hypnotic induction is the addition of the word hypnosis. Instead of saying ‘more and more deeply relaxed,’ the therapist says ‘more and more deeply hypnotised.’ Because relaxation training is a frequent component of behaviour therapy, the addition of hypnosis to behavior therapy may consist of little more that the use of the word ‘hypnosis.’” (Kirsch et al., 1995, p. 215)

Gandhi and Oakley (2005) were able to show 10 years later that it definitely makes a difference whether one uses the word “hypnosis” instead of the word “relaxation.” One can only speculate about the reasons for this systematic ignorance of hypnosis in today’s main-stream therapy which is cognitive behavior therapy. One of the reasons could be: Hypnosis fundamentally does not fit into the epistemology of cognitive behavior therapy, which is committed to enlightenment (Peter, 2023a) and does not refer to proto-therapeutic rituals such as exorcism or mesmerism, but emerged in the context of 20th century science.

### 2.4 Hypnosis and relaxation

Physical relaxation is often part of the ritual to induce hypnosis. The individual test items of the classic Stanford and Harvard hypnotizability scales, for example, are preceded by a hypnosis induction lasting around 20 min, which aims to induce a kind of sleep state via relaxation suggestions, which is then defined as hypnosis, e.g.: “I am about to give you some instructions that will help you to relax and gradually to enter a state of hypnosis. […] You are going to get much more drowsy and sleepy. Soon you will be deeply asleep […]” (Shor and Orne, 1962, p. 6). The reference to sleep has historical reasons. Since the terms “artificial Somanmbulism” (artificially induced sleepwaking) in the Romantic period and Braid’s (1843) “Neurypnology” (neurological sleep), the word “hypnosis” (Greek: sleep) has become established and has often had to be explained, e.g., that it has nothing to do with natural sleep (Evans, 1972).^3^ Nevertheless, the induction of good muscular relaxation makes perfect sense for a state of hypnosis, as it enables a reduction in muscular holding tension and thus the dissolution of the body ego, the “minimal phenomenal selfhood” (Blanke and Metzinger, 2009) as an introduction to the experience of what Weitzenhoffer (1974) called the “classic suggestion effect”: as long as someone has the experience of (healthy) bodily autonomy and experiences that he/she can raise his/her hand voluntarily as a physically active subject or “Ego,” he/she will not experience this as “hypnotic”—in contrast to the experience of involuntariness, i.e., that the hand raises by itself, although the body “Ego” is no longer consciously perceived. In the first case, the person follows an “instruction,” in the second case the person responds to a “suggestion” (Peter, 1996). Similar to this distinction between an arbitrary and involuntary motor response (Peter et al., 2012), a distinction can be made between voluntary imagination and hypnotic illusion or hallucination: As long as someone deliberately imagines what is suggested during a guided imagination and is aware that he/she is following the suggestions—and can decide in mental autonomy whether he/she wants to do so—it is called an imagination. However, as soon as the *sense of agency (SoA)* (Polito et al., 2014; Haggard, 2017), i.e., the sense of self, recedes and a person stops actively and arbitrarily imagining something, but only passively, involuntarily and uncritically sees, hears or feels what another person “suggests” (in the Latin sense of “slide underneath,” see above), the imagination becomes a hypnotic hallucination experienced as evident. Understood in this sense, hypnosis can therefore be defined as follows:

“Hypnosis can be defined as the art of creating an alternative reality by imagination, which, ideally, should be experienced like a hallucination or illusion. […] The more intense and real (evident) this alternative reality feels and the more it is experienced in form of hypnotic phenomena (i.e., hallucinations, illusions, and involuntary responses), the more likely normal reality is dismissed or dissociated, partially or as a whole, during the time of the trance state; and the more likely therapeutically relevant features of this alternative reality will eventually be implemented in everyday life. In that way, hypnotised patients can better tolerate aversive medical procedures, or, in the course of a hypnotherapy, can change their feelings, cognitions, and behavior.” (Peter, 2015b, p. 458)

A non-judgemental, involuntary acceptance of suggestions has always been regarded as a hallmark of hypnosis, but has often also been seen as a negative characteristic, namely a loss of control. Whether such a suggestive-hypnotically induced loss of control is also possible in normal everyday life, whether the hypnotized person is then helplessly at the mercy of the hypnotist, was extensively discussed in the 19th century (Liégeois, 1884) and repeatedly discussed thereafter (e.g., Barber, 1961; Orne and Evans, 1965; Peter, 2015c), but rather outside the psychotherapeutic and medical context. Within this therapeutic context, however, it can be helpful or even necessary for a patient to leave the psychopathological parts of their neurotic or psychosomatic “everyday personality” in order to gain new perspectives and have new emotional experiences, and thus temporarily allow a kind of “alien control.” In classical suggestive hypnosis, this was exclusively the therapeutic expertise of the psychotherapist, until within the framework of Milton H. Erickson’s hypnotherapy approach (Beahrs, 1977; Short, 2021), the old Romantic concept of the “unconscious” was reactivated as a metaphor for the patient’s positive unconscious resources, this time, however, not as a trans-personal concept as in German Romanticism at the beginning of the 19th century, but—in keeping with the times of humanism at the end of the 20th century—as an inter-personal “therapeutic tertium” (Peter, 2002).

### 2.5 Is there a special state of hypnosis or not?

Engaging with the positive experiences and the knowledge of the “unconscious” requires letting the everyday ego rest and not using its usual functions. Among hypnotherapists, this is usually referred to as conscious-unconscious dissociation, a key concept that Milton H. Erickson also advocated:

*“Deep hypnosis is the level of hypnosis that permits subjects to function adequately and directly at an unconscious level of awareness without interference of the conscious mind.”* (Erickson, 1952, p. 146, italics in original)

Dissociation refers to a basic and one of the oldest concepts in the history of hypnosis: In states of trance, after rituals of exorcism (Gaßner, 1774), animal magnetism (Mesmer, 1775), romantic somnambulism (Puységur, 1784) and finally hypnosis (Braid, 1843), people have always felt more or less clearly “dissociated” from aspects of their everyday personality and have accordingly also behaved more or less “dissociated.”

Formally, dissociation is understood as the separation or splitting off of psychic functions such as thoughts, feelings and experiences (and the associated behavior) that are normally experienced as belonging together or are integrated in the stream of consciousness. They define the physical and mental autonomy of a (healthy) individual. Uncontrolled, severe forms of dissociation are found in mental disorders (Kihlstrom et al., 1994); in a controlled form, dissociation represents the experience of hypnosis and hypnotic phenomena. Hypnotic phenomena correspond to psychopathological symptoms in phenomenological terms (*cf.*, Gruzelier et al., 2004), but differ from them essentially in that they are communicable and therefore controllable (Peter, 2023d).^4^

The phenomenon of dissociation was already described by Janet (1889) as *desaggregation* and further differentiated by Hilgard (1977) in his neo-dissociation theory as the splitting of ego functions, as well as by others who followed him (e.g., Bowers, 1991; Woody and Bowers, 1994; Jamieson and Woody, 2007). Since then, dissociation has been the basic concept of theories postulating hypnosis as a particular non-ordinary state of consciousness and, as illustrated, has been vigorously attacked by the sociocognitive non-state theorists (e.g., Barber and Wilson, 1977). The extent to which people can experience dissociation in the form of communicable and controllable hypnotic phenomena varies widely between individuals and is usually referred to as hypnotizability. This will be discussed in more detail below.

The following section presents experimental findings that support the hypothesis of hypnosis as a special state of consciousness.^5^ However, they were only obtained from highly hypnotizable persons, because only they are capable of this, not the low-hypnotizables. McGeown et al. (2009) and Mazzoni et al. (2013) showed that hypnosis induction had significant subjective effects with regard to visual hallucinations, but only a small effect on objective reactions; the decisive factor for the objective reaction was hypnotizability. Nevertheless, the authors emphasize the role of hypnosis induction: it helps highly suggestible persons to better focus their attention and cognitive resources on the respective hypnotic task. However, the significant factor in the study of Mazzoni et al. (2013) was a “hidden” special experimental condition with which the authors were able to prove that there is actually a special state of consciousness that could be assigned to hypnosis. The previously notorious non-state theorist Irving Kirsch could not help but “to reconsider my position on the altered state issue” (Kirsch, 2011, p. 355). The highly suggestible^6^ subjects not only reported that they felt hypnotized after a hypnosis induction, but they also demonstrated the effect of the induction neuro-physiologically: During the rest periods between the hallucination tasks, they showed reduced activity of medio-prefrontal parts of the *default mode network (DMN)* (Raichle, 2015).^7^ This deactivation of the *DMN* correlated both with the subjectively perceived depth of hypnosis and with the clarity of the visual hallucinations: the lower the activity in the *DMN*, the deeper the subjects felt in hypnosis and the more clearly they perceived the positive or negative hallucinations (during the visual test tasks). The low-suggestible subjects, on the other hand, did not show this deactivation in the *DMN* but in the thalamus, which indicates that they were simply relaxing. This deactivation of the *DMN* in the highly suggestible subjects after a hypnosis induction, which was also found in other hypnosis studies, for example by Deeley et al. (2012) or McGeown et al. (2015), could now be interpreted as a signature of the hypnotic state, because it “creates a distinctive and unique pattern of brain activation in highly suggestible subjects that is different from those observed in low suggestible people” (Mazzoni et al., 2013, p. 405).

These results were confirmed by Jiang et al. (2017), who found reduced coupling between the dorsolateral prefrontal cortex (DLPC), which is part of the executive network, and parts of the parietal parts of the DMN, the posterior cingulum (PCC): “This reinforces the notion of hypnosis as a different state of consciousness rather than a reduced level of arousal” (Jiang et al., 2017, p. 4091). These *DMN* results confirm the hypo-frontality hypothesis (Dietrich, 2003). The reduced activity of the *DMN* could be interpreted that during hypnosis, those cognitive activities that deal with self-referential considerations about oneself and with evaluative examination of the self-image are omitted in highly hypnotizable people. This deactivation especially of the medio-frontal *DMN* in particular would make the reduction in empathy understandable (*cf.* Damasio, 1994), which would explain the socially unacceptable behavior in stage hypnosis (Parris, 2016). “This means that the hypnotized person experiences a reduced representation of the everyday ego as well as an altered body representation and is focused with their attention exclusively on suggestions or ideas and not just relaxed and sleepy” (Revenstorf, 2023, p. 44). However, this “reduced representation of the everyday ego” is only one of the possible interpretations of the *DMN* results. Another interpretation states that the *DMN* reduction indicates that the subjects interpreted the hypnosis situation as a goal-oriented task of normal everyday life:

“Decreases in default-mode activity are associated with increased goal-directed activity in everyday life and are therefore also consistent with our hypothesis that goal-directed, strategic, and possibly *nonconscious mental activity can play a role in hypnotic responding*, much as it does on a day-to-day basis.” (Lynn et al., 2015, p. 322, italics added)

However, this acknowledgement to a “non-conscious mental activity” can in turn be understood in the sense of a reduced activity of the everyday ego and would then again be in line with the hypo-frontality thesis. With the reduced activity of the *DMN*, an objective correlate for hypnosis would be given, which, however, requires high hypnotizability as a special intra-personal characteristic and is therefore only applicable to the highly hypnotizable, i.e., to about a quarter to a maximum of a third of the patients or probands. Hypnotizability in these individuals is obviously related to a special, neuro-physiologically detectable state of consciousness during hypnosis (see also Kallio and Revonsuo, 2003).

The reported brain imaging studies were conducted on the basis of sensory, especially visual phenomena, which are interesting in themselves (*cf.* also Kosslyn et al., 2000). Even without a relationship to the DMN, changes in the brain have also been demonstrated for acoustic (Szechtman et al., 1998) and motor phenomena (e.g., Cojan et al., 2009; Pyka et al., 2011; Burgmer et al., 2013), which make the decoupling or disconnection of brain areas that normally, i.e., without hypnosis, interact visible. These studies confirm the dissociation theories of hypnosis. The changes in the brain caused by hypnosis have also been demonstrated using event-related brain potentials, e.g., for the most important sensory-affective phenomena such as analgesia (as a kind of negative kinesthetic hallucination) (Miltner and Weiss, 2007; Franz et al., 2024), for negative acoustic (Franz et al., 2020) and for negative visual (Franz et al., 2021) hallucinations, as well as for purely cognitive phenomena such as post-hypnotic suggestions (Zahedi et al., 2020; 2023b).

These studies also show that hypnotic phenomena are based on top-down regulations that are able to overwrite bottom-up signals of current sensory input (Landry et al., 2017; Terhune et al., 2017), but only in high hypnotizables. This may lead to a shift from left-hemispheric to right-hemispheric processes—again, only in high, not in low hypnotizables—as pointed out by Gruzelier (2004) and confirmed by Naish (2010) or Lanfranco et al. (2021). It remains to be seen how much enlightenment the latest hypnosis theory by Martin and Pacherie (2019) and Zahedi et al. (2023a), which is based on the predictive coding model of Friston (2018), will provide.

### 2.6 Hypnotizability

People experience the two criteria of hypnosis, involuntariness and evidence (Peter, 2015b), to varying degrees; involuntariness and evidence define the change in the *sense of agency (SoA)* that determines the subjective experience of hypnosis. The more or less pronounced intra-psychic ability to do this is referred to here as hypnotizability.

Mesmer’s predecessor Gassner (Peter, 2005), Mesmer himself and many of his successors in the history of hypnosis already described differences in their patients’ ability to follow the given suggestions (Peter, 2024). Hypnotic receptivity, hypnotic suggestibility or hypnotizability has been systematically researched since Hull (1933) and Hilgard (1965). It is a personal characteristic that remains stable over the lifespan (Morgan et al., 1974). Whether the normal distribution (10–25% highly hypnotizable, 10–25% low hypnotizable and the rest more or less hypnotizable) found in numerous studies, predominantly on student populations, is representative of the general population has yet to be proven (Peter and Roberts, 2022). For example, when using a shortened version of the HGSHS-5:G (Riegel et al., 2021), which comprises only 5 instead of the 12 items of the HGSHS:A (Shor and Orne, 1962) and thus appears to be significantly shorter and much more suitable for clinical studies, skewed score distributions were observed: a right skew (more high hypnotizables) in participants of a hypnosis congress (Wolf et al., 2022), and a left skew (fewer high hypnotizables) in patients. These results are analyzed in more detail in another article in this Research Topic (Zech et al., 2024). Correlations with other personality traits are uncertain. However, “hypnophilic” people, i.e., people who are generally interested in hypnosis, show high levels of the schizotypal personality style, the more so the more hypnotizable they are (see also Jamieson and Gruzelier, 2001). A whole series of studies indicate that we can speak of a *homo hypnoticus* in this context (Peter et al., 2014; Peter and Böbel, 2020; Wolf et al., 2022; Peter and Wolf, 2023).

Hypnotizability usually is tested after a hypnosis induction by presenting hypnotic phenomena of varying difficulty. Phenomenologically, the hypnotic phenomena can be categorized into four groups (Peter, 2023d):

1. The direct motor-kinesthetic phenomena, which are based on the relaxation of the musculature such as lowering the head or outstretched arm, can be realized by almost all people. More difficult are the active motor-kinesthetic phenomena that require an involuntary increase in muscle tone, such as arm levitation (Blakemore et al., 2003; Peter et al., 2012, 2013). And even more difficult, i.e., realizable by even fewer people, are the motor “challenge” phenomena when it is suggested that, for example, an outstretched arm can no longer be bent arbitrarily or a hand resting in the lap can no more be lifted (Cojan et al., 2009; Pyka et al., 2011). The criterion of *sense of agency* (*SoA*) plays a decisive role in involuntariness, because authorship also means in particular being able to use one’s own skeletal muscles voluntarily.
2. The criterion of evidence is important for sensory-affective phenomena. These affect all five senses and should be experienced as evidently as possible, i.e., in the sense of positive or negative visual (Kosslyn et al., 2000), acoustic (Szechtman et al., 1998), kinesthetic (proprio- and interoceptive) (Rainville et al., 1997; Derbyshire et al., 2004; Raij et al., 2005), olfactory or gustatory hallucinations or illusions.
3. Purely cognitive phenomena refer to amnesic phenomena and posthypnotic suggestions (Kihlstrom, 2021; Zahedi et al., 2023a). (Usually the sensory-affective phenomena are not presented separately, but as a common group with the cognitive phenomena.^8^)
4. *Sense of agency* (*SoA*) as well as involuntariness and evidence are particularly relevant for identity delusions, which are not included in the known hypnotizability scales, presumably because they are only mastered by very few highly hypnotizable people, so-called *hypnotic virtuosi*, and could be dangerous for vulnerable people (Revenstorf and Peter, 2023). They concern more serious changes in the “ego” identity, i.e., modelling psychotic or neurological disorders, but are definitely shown in stage hypnosis, which sometimes leads to serious problems (e.g., Kleinhauz et al., 1984; Gruzelier, 2004). These phenomena have been systematically investigated by an Australian research group (e.g., Connors et al., 2015; Coltheart et al., 2018).

Around 80–90% of a sample can successfully master the motor-kinesthetic test items, only 10–20% can demonstrate the cognitive phenomena, even fewer the identity delusions, and the remainder respond more or less well to the sensory-affective phenomena. According to the number of tasks solved, they are usually divided into low, medium and high hypnotizables. The classic hypnotizability tests use dichotomous scoring based on the criteria of involuntariness and evidence (e.g., the hand must have actually lifted during the arm levitation, imagination alone is not sufficient). The more recent Elkins Hypnotizability Scale (EHS) (Elkins, 2017), on the other hand, allows a more differentiated assessment by not requiring the criteria of involuntariness and evidence to be absolute, but also accepts imaginative representations of the tasks, but scores them lower. Another advantage of the EHS is that, at approx. 30 min, it requires only half as much time as the classic Stanford and Harvard scales (SSHS, Weitzenhoffer and Hilgard, 1959; HGSHS, Shor and Orne, 1962).

Analogous to the scientific discourse on the question of whether hypnosis is a special state of consciousness or merely the result of culturally shaped social interaction, a series of studies were conducted in the 1980s and 1990s on the question of whether hypnotizability is actually a stable, genetically determined trait (Morgan, 1973) or whether it can also be trained as a normal human ability, as Spanos et al. (1983) attempted to prove. Today, the result of these studies can probably be summarized as follows: hypnosis training certainly helps those with low hypnotizability, but is of no benefit to those with high hypnotizability, as reconfirmed by Rasch and Cordi (2023, this issue).

Hypnotizability is a patient variable that is unique to measure in psychotherapy, as there are no indication instruments for other psychotherapeutic procedures such as psychoanalysis or behavioural therapy. Analogous to the classical pharmacology model, it was long assumed in psychotherapy that a particular psychotherapeutic procedure was equally effective for all patients. This attitude is about to change in general psychotherapy (*cf.* e.g., Beutler et al., 2016; Heinonen et al., 2022), but has also only been partially taken into account in hypnosis. In experimental hypnosis research, the measurement of hypnotizability is standard, because only in this way can the suggested effects be related back to hypnosis under controlled conditions. In clinical research, on the other hand, and even more so in hypnotherapeutic practice, such measurements are usually neglected. In an international survey of professionals using clinical hypnosis, only 26.6% rated hypnotizability as “important or extremely important for therapeutic success” (Palsson et al., 2023, p. 104). The reason often given is that this measurement could be an additional burden for the patients or that it would contribute little to the clarification of variance anyway. In a first meta-analysis of studies on hypnotic analgesia, Montgomery et al. (2000), for example, found a significant correlation between hypnotizability and effect (effect sizes for high-hypnotizables d = 1.22, for medium-hypnotizables d = 0.64, for low-hypnotizables d = 0.10). However, Montgomery et al. (2011) relativized these impressive figures in a later meta-analysis of randomized clinical trials (RCT): although there was a significant relationship between hypnotizability and treatment effect, hypnotizability only contributed 6% to the variance explanation. However, of the 10 studies included, only three related to mental disorders, and with small numbers of patients (N = 32, 20 and 24) in the hypnosis groups. In general, this sheds light on the still precarious situation of psychotherapy-relevant hypnosis research, but also calls into question the authors’ conclusion, which some clinical hypnosis researchers refer to when they claim that hypnotizability does not need to be measured: “The results […] raise the question of the overall utility of assessing hypnotic suggestibility in clinical settings” (Montgomery et al., 2011, p. 303). The conclusion should rather be: There is a need for (1) considerably more psychotherapy-relevant studies that (2) meet today’s standard RCT criteria and (3) have such large numbers of patients that the patient variable hypnotizability becomes visible as a moderator alongside the many other therapy variables. In the most recent RCT by Fuhr et al. (2021) with certainly more patients (n = 78 and 74), hypnotizability was measured but unfortunately not related to the effect of the hypnotherapy used for depression [which was as good as the gold standard treatment for depression, cognitive behavior therapy, which was still evident after three and a half years (Fuhr et al., 2023b)]. The most recent study by this research group (Fuhr et al., 2023a, this issue), on the other hand, had far too few patients to meaningfully measure hypnotizability. Due to a lack of data, hypnotizability was unfortunately also not taken into account in the meta-analysis by Milling et al. (2019), in which a mean weighted effect size of 0.71 was found in 13 studies on the treatment of depression with hypnosis, suggesting that the average participant receiving hypnosis showed more improvement than about 76% of control participants.

Until new meaningful studies are available, psychotherapists working with hypnosis can only report from their subjective experience that the hypnotizability of their patients in the course and outcome of hypnotherapy is of relevance that should not be underestimated, because it brings real benefit for many of those who are moderately hypnotizable, but above all for those who are highly hypnotizable.

However, hypnotizability plays a significant role not only within hypnotherapy, for example in the proven functional equivalence between imagination (i.e., hallucination) and perception in highly hypnotizable individuals (Santarcangelo et al., 2010; Ibáñez-Marcelo et al., 2019). According to Malloggi and Santarcangelo (2023), this functional equivalence could also lead to better results of imagination training in neurological patients. According to these authors, the special type of information processing of the highly hypnotizable could also result in greater resilience to brain injuries; their more adaptive cardio- and cerebrovascular functions could predict a lower susceptibility to vascular events and enable the personalization of pharmacological pain therapies.

## 3 Hypnosis in medicine

The therapist variable “hypnotization ability,” i.e., the ability to induce hypnosis convincingly and to work with explicit hypnotic phenomena, has not yet been researched at all. This is especially true also for the application in medicine, all the more so as communicative and interactional techniques and strategies are rarely part of the medical training, but are essential how the doctors talk to their patients. Especially if one does not want to carry out explicit and time-consuming hypnosis inductions in medicine, but rather strives for a broad use of special “hypnotic” or “suggestive” communication and interventions, e.g., the suggestion of a dissociation to a safe place and the reframing of disturbing noises for surgery under local or regional anesthesia, it is important to offer this to all patients without pre-testing, even if the effect may vary. In this sense, “hypnosis” is becoming increasingly important in medicine.

Generally, the hypnosis research situation in medicine is better than in hypnotherapy. The current scoping review by Hagl (2023a), for example, lists 11 RCTs on acute medical interventions for the year 2022; among the 14 RCTs on chronic complaints, only five RCTs relate to psychological complaints or behavioral problems (test anxiety, smoking cessation, withdrawal symptoms, obesity), while the remaining nine relate to purely medical problems. Here, relatively short interventions (up to three sessions) were often used or even just audios for self-hypnosis without therapeutic contact. The detailed meta-analysis by Rosendahl et al. (2023) over the last 20 years confirms this impression. It is obvious that the time, personnel and financial costs involved in medical hypnosis examinations are much more manageable and the examination conditions much easier to control than in psychotherapeutic ones.

The classic and probably best-researched area of application for medical hypnosis is pain. Before the introduction of ether in 1846 and chloroform in 1847, the proto-form of hypnosis, mesmerism, had already been described in detail as a successful technique by Elliotson (1843) and Esdaile (1846), but was only intensively researched more than 100 years later by Ernest R. Hilgard and others (Hilgard and Hilgard, 1975). Even today, hypnosis is still an important field of application for pain therapy (Erickson, 1967; Jensen, 2011; Peter, 2019), which is reflected in scoping reviews (Pathak et al., 2020) and meta-analyses (Montgomery et al., 2000; Thompson et al., 2019; Milling et al., 2021; Merz et al., 2022). Hypnosis (Häuser et al., 2016) or therapeutic communication based on hypnotic principles can also be effective during unpleasant medical procedures. The extensive research by Elvira Lang (e.g., Lang et al., 1996, 2021) or Elisabeth Faymonville (e.g., Faymonville et al., 1997, 1999) and Ernil Hansen (e.g., Hansen et al., 2023) has become well known in this regard. The most recent meta-analysis by Holler et al. (2021) about hypnosis in adults undergoing surgical procedures also confirms the positive effects of hypnosis. The studies confirm the practical experience in everyday clinical practice:

“The adjunctive use of hypnosis before, during or after a surgical or diagnostic procedure with local or general anesthesia reduces both the emotional stress of the procedure and the associated pain. It also reduces the use of medication and shortens the duration of surgery and convalescence, in each case with small to medium effects over and above standard medical treatment.” (Hagl, 2023b, p. 756 f)

A cost analysis also shows a considerable savings potential through the adjunctive use of hypnosis: “the cost associated with standard [i.e., intravenous conscious] sedation during a procedure was $638, compared with $300 for sedation with adjunct hypnosis, which resulted in a savings of $338 per case with hypnosis” (Lang and Rosen, 2002, p. 375).

In any case, the success of hypnotic interventions in medical contexts has been well established by a number of meta-analyses (summarized in Hansen, 2023b). Hypnosis is also used in dentistry (Schmierer and Wolf, 2023) or in obstetrics (Hüsken-Janßen and Fisch, 2023). Franch et al. (2023) showed by a systematic review that hypnosis improves symptoms caused by oncological interventions and the disease itself when used by qualified professionals as an adjuvant to well-established treatments.

An extreme example of how successfully hypnotherapeutic interventions and communication can be used in surgical procedures under local or regional anesthesia, i.e., with the patient awake, is awake craniotomies, where the patient must be awake at least temporarily during brain surgery for intraoperative testing. While the standard procedure for deep brain stimulation or resection of a tumor in the vicinity of eloquent brain areas is an alternating sequence of anesthesia or deep sedation with awake phases (sleep-awake-sleep-technique), patient can stand these operations of 5–6 h by means of hypnotic communication without anesthetics with the according advantages under active participation (Hansen et al., 2013; Zech et al., 2018). EEG changes measured with a monitor for anesthetic depth have been documented like they are observed during pharmacological sedation or after inductions of hypnosis (Zech et al., 2023). Since hypnotic communication routinely works in this indication to avoid and to largely dispense analgesics, a muscle biopsy or tooth extraction should not pose a problem. Meanwhile also use of classical hypnosis has been reported in this indication (Frati et al., 2019).

There are many obstacles on the path of hypnosis back into medicine, some of which stem from the different paths the two have taken. Hansen (2023a) has analyzed the difficulties and made suggestions for overcoming them: Supported by well-designed studies, a more rigorous scientific evidence-base is needed. Hypnosis should be represented in publications in recognized medical journals with high impact and accessibility, in medical congresses discussing clinical care, and in treatment guidelines. In addition to being an exceptional treatment for selected patients, hypnosis in medicine could allow better care for all patients in everyday health care.

### 3.1 Is hypnosis induction necessary for medical applications?

Despite the obvious advantages, hypnosis has not been—and is still not used enough in medicine. We can only speculate about the reasons for this. One of these reasons is certainly a very pragmatic one, namely the duration of hypnosis inductions. These take time, which is generally not available in everyday medical practice. The induction suggestions in the classic hypnotizability scales, for example, last up to 20 min. For most people, this is the usual amount of time for profound physical relaxation so that, ideally, their body ego, their “minimal phenomenal selfhood” (Blanke and Metzinger, 2009), can dissolve and, as a result, their *sense of agency (SoA)* can change. This changes the usual everyday consciousness with its evaluative, task- or ego-centered orientation and creates focused attention, which is a prerequisite for uncritically accepting suggestions. This can easily be done in a psychotherapeutic practice. But does this also apply to medical contexts? Elvira Lang, Marie-Elisabeth Faymonville and Ernil Hansen have shown that good effects can also be achieved without prior explicit hypnosis induction. Recalling the article “Importance of recognizing that surgical patients behave as though hypnotized” by the American gynecologist Cheek (1962a)—who, incidentally, was the first to report on “unconscious perception of meaningful sounds during surgical anesthesia as revealed under hypnosis” (Cheek, 1959)—Ernil Hansen assumes that no explicit induction of hypnosis is necessary for doctors, let alone in hospitals, because patients in such situations are already in an altered state of consciousness that is similar or identical to the hypnotic “trance.” Firstly, trance is regarded, e.g., by Hansen and Zech (2019) as a natural ability of every human being, which occurs spontaneously as a “natural trance” in a regular (ultradian) rhythm (Rossi, 1991). Secondly, the ability to trance is seen as a biological emergency and protective reaction that enables the organism to access physiological functions anchored in the unconscious such as analgesia, vasoconstriction, dissociation, catalepsy (dead man’s reflex), amnesia and much more (Hansen and Zech, 2019). Accordingly, especially in acute medical situations such as at the accident site, in an operating room, a dental chair, the intensive care unit, an irradiation room, the delivery room and many more, hypnosis-experienced medical staff can regularly observe trance signs in patients (Hansen and Bejenke, 2010). This is plausible insofar as in such situations a person’s self-efficacy is reduced to the point of absolute helplessness and they are dependent on effective actions and instructions (“suggestions”) from others. If voluntary competence to act is reduced and attention is highly focused, this corresponds to one of the prerequisites of hypnosis, namely motor restriction and sensory deprivation (Peter, 1994, 2023e), and such situations possibly produces a state of situational hypersuggestibility (Hull, 1933): Patients at the doctor’s and especially in hospital are highly receptive to all information, positive and negative, placebo and nocebo suggestions (Hansen and Zech, 2019; Zech et al., 2019, 2020, 2022), this even applies to operations under general anaesthesia (Nowak et al., 2020, 2022).

Whether this situational hypersuggestibility correlates with high hypnotizability and/or even requires a special hypnotic “trance” state requires further investigation, just as the relationship between hypnotizability and suggestibility in general is still open to further research. This is because the ability or willingness to respond to suggestions in an *inter-personal* context is completely independent of hypnosis and is called suggestibility—in contrast to hypnotizability as an *intra-personal* variable. Suggestibility in general is the ability to react spontaneously to suggestive communication without checking its content for accuracy or possible alternatives. Such general suggestions do not require hypnosis to be effective. This non-hypnotic, imaginative or waking suggestibility also includes sensory and interrogative suggestibility as well as placebo reactions. This was already noted by Bernheim (1886), discussed in detail by Sidis (1898), Binet (1900), Straus (1927) or Stokvis (1957) and later analyzed in detail by others (e.g., Gudjonsson, 1987; Gheorghiu et al., 1989; Loftus and Banaji, 1989; Gheorghiu, 2000). The fact that people are hypersuggestible, especially in emergency and many medical situations, was already described at the beginning of the 20th century: “Hypnosis […] can be induced by *choking or fright*, − in the case of serious accidents or violent natural events, individuals show lack of judgement, paralysis, painlessness and will-less obedience” (Bergmann, 1912, p. 139, emphasis in original). It is interesting that Bergmann refers to this phenomenon of increased suggestibility as hypnosis, because even today people still occasionally speak of spontaneous (auto-) hypnosis or (problem-) trance in this context, which may sound plausible at first, but is not entirely correct in terms of conceptual theory: hypnosis does increase suggestibility (Braffman and Kirsch, 1999), but not everything that increases suggestibility is hypnosis. Non-hypnotic suggestibility (Oakley et al., 2021) and placebo responses in particular (Kirsch, 2019) are important topics, especially in medical contexts, which have nothing to do with hypnosis and are examined separately.

### 3.2 Hypnosis and trance

Hansen and Zech (2019) use the term “trance” in the context of medical hypnosis as a matter of course and are thus in good company with other “hypnophilic” (Peter and Böbel, 2020) professionals. The term trance has become commonplace in German-language literature in particular—“Trance and the objectives of hypnotherapy” (Revenstorf, 2023)—but is avoided in Anglo-American literature because it is too opaque, undifferentiated, even dangerous—“The myth of trance is arguably the mother of all myths and has birthed many related myths” (Lynn et al., 2020, p. 1254)—because it reinforces many of the popular misconceptions about hypnosis that still exist. Lynn et al. agree here with Nicholas Spanos (1986), who, for example, also used the term “trance logic” introduced by Martin Orne (1959) only in quotation marks and criticized it extensively from his socio-cognitive non-state position.

Trance is in fact a very general term that stands for many subjective experiences. Colloquially, the term “trance” or “trance”-state refers to persons not fully reality-oriented in certain situations, or, put differently, are introverted or “absorbed” by something specific (Tellegen and Atkinson, 1974). The situations in which people appear “as if in a trance” can be very diverse. One speaks of dance trance, religious or ecstatic trance, highway trance, conversational trance, everyday trance or when someone simply looks around thoughtlessly. The term trance therefore always requires a context-specific definition. Whether people in such states are particularly open, receptive or suggestible to external information or suggestions depends on the social context, i.e., whether the person is in contact and communication—in “rapport”—with another person. Such trance states can be used for both positive and negative suggestions, intentionally or unintentionally. In clinical contexts, one also speaks of problem trances when people are completely thrown back on themselves and unresponsive to others because they are helpless or anxious, e.g., in special medical situations. It is then necessary to re-establish contact and communication and offer constructive, helpful suggestions. In all these and similar situations, however, no explicit hypnosis induction has taken place beforehand, which is why it is problematic to use the term trance in general in connection with hypnosis. At the very least, the adjective “hypnotic” should be added to indicate that the trance took place after a hypnotic induction or at least in an explicit hypnotic context.

## 4 Resume

Hypnosis has a long history in which its “nature” has repeatedly been the subject of fierce controversy, so that many researchers and clinicians, especially natural scientists, have regarded it at best as an interesting arcanum and at worst as a negligible esoteric fringe phenomenon confined to lay healers and stage hypnotists. As an adjuvant to other psychotherapeutic methods such as psychodynamic or cognitive-behavior therapy, it has occasionally received recognition. The Scientific Advisory Board for Psychotherapy in Germany (WBP, 2006), e.g., has scientifically recognized hypnotherapy as a “method,” which is in line with the Mainstream view of the international hypnosis community, as Erika Fromm stated in an interview in 1998: “hypnosis is not, in and of itself, therapy […] it is a tool to me and I would like to keep it there” (Peter, 1998). This assessment of hypnosis as a tool is certainly true for the use of hypnosis in medicine, where hypnosis has only an auxiliary function for actual medical treatment, but this does not have to be applied to psychotherapy. At least the Milton Erickson Society for Clinical Hypnosis, Germany (M.E.G.) has sought recognition for “hypnotherapy” as an independent psychotherapeutic approach. However, this is still pending, as the necessary relevant studies are lacking.^9^ The situation is different for medical hypnosis where there are meanwhile sufficient clinical studies, but so far with little impact in everyday medical practice. The situation for clinical hypnosis as a whole is therefore still unsatisfactory. It remains to be seen how well a new generation of clinicians and researchers will be able to change this.

## 5 Outlook

It will be interesting to follow the latest developments with virtual reality technologies and their impact on hypnosis. The results to date are as yet inconclusive (*cf.* Rousseaux et al., 2020, versus Terzulli et al., 2023). Whether virtual reality alone or in combination with hypnosis can replace the social contact of the therapeutic alliance is to be seen (Saffy et al., 2023). The same applies to the use of artificial intelligence, which is expected to be used in hypnosis soon. Can we perhaps do without personal therapeutic contact in the psychotherapy of the future? Let us look back: Mesmer’s patients supplied themselves with the healing fluid from the Baquet, which had previously been magnetized by Mesmer himself, via iron rods or (in the back rows) via ropes; Puysègur’s patients did the same with hemp ropes hanging from a magnetized lime tree. The fact that the healing did not actually come about through these healing devices, but through the imagination of the patients—or through their expectations, as Kirsch (2000) would say—was already scientifically proven in 1784. There are good reasons to assume that the same psychic forces will play a role in the future.

It should also be considered that psychotherapeutic knowledge of change has improved significantly over the last 250 years both within and especially outside of hypnosis: psychoanalysis, behavior therapy and the many approaches of humanistic psychotherapy have been developed after hypnosis. Simply relying on the power of expectation, the self-healing powers of the organism or the wisdom of the unconscious via powerful suggestions or imaginations—with or without hypnosis—would be naïve and no longer meets today’s standards of psychotherapeutic professionalism, which takes into account the indication or function of a given hypnosis intervention. For the purpose of anxiolytic relaxation before a medical procedure and/or the pain-relieving effect of special imaginations, it is probably irrelevant whether the hypnosis induction is given live or tape-recorded, because the difference is marginal at best, as Lush et al. (2021) and Rousseaux et al. (2022) have demonstrated. For more complex psychotherapeutic indications such as personality disorders or chronically fixed symptoms, however, it is not enough to induce a hypnotic trance and then give ego-strengthening suggestions, tell trance-inducing stories or simply invoke the wisdom of the unconscious. Such disorders still require, and will certainly continue to require, specialized psychotherapeutic expertise, as Milton H. Erickson (Erickson and Rossi, 1979) or Janet (1889, 1897) have shown a long time ago. Both Janet and Erickson are known to have made a continuous effort to engage in deliberate practice (Tracey et al., 2024) (with or without hypnosis) and not simply rely on their experience. Thus, hypnotherapy will also have to develop further and this will continue to challenge the creativity of real human beings. Most recently Wilhelm-Gößling et al. (2020) have created the highly differentiated hypnotherapy manual for the RCT depression study by Fuhr et al. (2021). Such patients will presumably also require a good therapeutic rapport in the future, generally in psychotherapy (Heinonen et al., 2022) and also in hypnotherapy (Peter and Revenstorf, 2018; Varga, 2021), where therapists ideally also should be sensitive to the patients’ attachment experiences (Varga and Kekecs, 2014; Egozi et al., 2023; Di Filippo and Perri, 2024). Even in remote online therapy, which was frequently practiced during the Covid pandemic, users with secure attachment showed online a better therapeutic alliance than those with insecure attachment (Mercadal Rotger and Cabré, 2022). Whether avatars equipped with artificial intelligence are able to achieve this is questionable (Grodniewicz and Hohol, 2023), as is whether these avatars can adapt to the respective hypnotizability of their patients. It can be assumed that there will continue to be a certain number of people who are highly hypnotizable and whose neurophysiological make-up goes beyond the effects of expectation and imagination. Highly hypnotizable people will therefore remain to be of interest not only to science (e.g., Santarcangelo, 2024) but also to medicine because of the heightened suggestibility of patients. Hypnotizability probably also plays a greater role in psychotherapy in general than is usually assumed. In hypnotherapy in particular, highly hypnotizable people have to be told explicitly that they should go into hypnosis and not simply relax (Gandhi and Oakley, 2005). The traditional instruction/suggestion for this is a hypnosis induction. Hypnosis inductions are not useful to low-hypnotizables, whereas they are certainly beneficial to medium-hypnotizables. The latter however need a more intensive hypnotic rapport (Lynn et al., 1991) as well as more elaborate suggestions (Szabo, 1996), such as those taught in Ericksonian hypnotherapy. They may also benefit from virtual reality (Engelhardt et al., 2019).

Hypnosis has been part of the Western world’s social culture for around 250 years. Although there were some misconceptions about its nature and possibilities, the general population’s opinion of hypnosis seems to be positive or at least neutral (Palsson et al., 2019). In terms of scientific recognition, hypnosis has experienced turbulent ups and downs during this 250-year history. Since around the middle of the 20th century, hypnosis has become the subject of serious scientific investigation, but mainly in its experimental form. As shown, there is still much room for improvement in the scientific evidence of the effectiveness of clinical hypnosis in psychotherapy and psychosomatics as well as its practical application in medicine.

## Author contributions

BP: Writing – original draft, Writing – review & editing.

## Footnotes

^1^The idea that sensory deprivation and motor restriction are essential situational characteristics of hypnosis goes back to Christoph Kraiker: “Hypnosis produces a comparable state of sensory and motor deprivation; the imaginative processes are suggestively well controllable, and thus reconditioning can be achieved that could not be established by interference from external or internal disturbing stimuli or by motor auto-stimulation” (Kraiker, 1991, p. 194; *cf.* also Kraiker, 1985,^1992^).

^2^A similar idea had already been described by Zarbock (1994), using hypnosis in combination with cognitive behavior therapy. (I am thankful to Wolfram Dorrmann for this hint.)

^3^Even the suggestion of relaxation does not necessarily have anything to do with hypnosis, as Wilson and Barber (1978) or Banyai and Hilgard (1976) have shown.

^4^Although the arm rises involuntarily (“as if by itself”) during an armlevitation, it reacts to the verbal request (“suggestion”) to rise; and it lowers again after the corresponding verbal suggestion. If it were to remain cataleptically in the air and no longer react to a verbal suggestion, the hypnotic phenomenon of armlevitation would have “transformed” into the dissociative symptom of paralysis.

^5^Instead of “special,” the adjective “non-ordinary” is used more frequently today to describe the state of hypnosis. This certainly makes sense in order to put the old dispute between the special state and the non-state followers to rest. In this article, both terms are used interchangeably.

^6^In the literature, the adjectives “suggestible” and “hypnotizable” are often used synonymously.

^7^The DMN consists of medial-prefrontal (ventral and dorsal medial prefrontal cortex) and medial-posterior areas (precuneus and posterior cingulate) (Raichle et al., 2001) and is activated when the brain is not occupied with external goals and tasks, but with “intrinsic” or “task-negative” activities. In contrast to the “extrinsic” or “task-positive” areas, the DMN is associated with self-referential, ego-related functions and autobiographical memory; it is, so to speak, the “echo chamber” of our ordinary everyday ego, a kind of “neural self” that is active when we have nothing to do but occupy ourselves with ourselves, daydream, think about our present, past or future, but also speculate about what others might be feeling and thinking (empathy and theory of mind).

^8^As one of the few exceptions, Woody et al. (2005) differentiated between “Perceptual-Cognitive” and “Posthypnotic Amnesia” items.

^9^“Hypnosis psychotherapy” is institutionally recognized in Austria. State recognition of hypnosis or recognition by health insurance companies varies greatly from country to country. Further information can be obtained from www.ishhypnosis.org or info@ishhypnosis.org.
